# Socio-economic, demographic, and contextual predictors of malnutrition among children aged 6–59 months in Nigeria

**DOI:** 10.1186/s40795-023-00813-x

**Published:** 2024-01-02

**Authors:** Phillips Edomwonyi Obasohan, Stephen J. Walters, Richard Jacques, Khaled Khatab

**Affiliations:** 1 Sheffiield Centre of Health and Related Research (SCHARR), A Division of Population Health, University of Sheffield, Sheffield, S1 4DA UK; 2 Department of Liberal Studies, College of Administrative and Business Studies, Niger State Polytechnic, Bida Campus, 912231 Bida, Nigeria; 3 Faculty of Health and Wellbeing, Sheffield Hallam University, S10 2BP Sheffield, UK

**Keywords:** Double burden of malnutrition, Predictors, Stunting, Wasting, Underweight, Overweight, Under-five years, Nigeria

## Abstract

Malnutrition has remained a global public health issue, particularly in low- and middle-income countries (LMICs). Researchers have committed to studying malnutrition (especially in children under the age of five) to address the nine malnutrition targets, set by the WHO to be achieved by 2025. This study seeks to evaluate the prevalence, the individual and contextual predictors of malnutrition among children aged 6–59 months across Nigeria and its states. Two separates, independently collected, nationally representative cross-sectional surveys, the National Human Development Report (NHDR 2018) and the 2018 Nigeria Demographic and Health Survey (2018 NDHS) were linked for this study. Spatial map was used to describe the prevalence of malnutrition, a 3-level multivariate multilevel logistic regression models were fitted where children/individuals (at level 1) were nested in communities/clusters (at level 2) and nested in states (at level 3). A weighted sample of 7,770 children 6–59 months were considered in this study. The results showed that an estimated 43.6% of children aged 6–59 months are poorly nourished in Nigeria. The proportions of poorly nourished children were generally highest in the Northern Nigeria. Child’s gender, age, birth size, preceding birth order, anaemia status, maternal education, work status, body weight, household wealth status, number of bedrooms were among individual/household predictors of malnutrition. On the community level, being from community with high wealth index, distance to nearest health facilities is no big problem. Regional variations and gender inequality index were the state level predictors of malnutrition among children in Nigeria. This study has shown that two-third of children aged 6–59 months in Nigeria were poorly nourished, an indication of a growing concern of double burden of malnutrition in Nigeria.

## Introduction

Malnutrition which “refers to deficiencies, excesses, or imbalances in a person’s intake of energy and nutrients” [1], has continued to be a public health concern world over and especially in developing countries [2]. Over 200 million children under-five are either undernourished or overweight [1]. Undernutrition in children is contributing about 45% of under-five mortality worldwide [1]. The World Health Organisation (WHO) member states recently ratified a commitment to nine global targets by 2025, including a 40% decrease in childhood stunting, a prevalence of childhood wasting of less than 5%, a guarantee that the number of overweight children will not rise, and the elimination of all forms of malnutrition by 2030 [3]. With less than three years till the goal date, the development has remained minimal, and none of the nine targets have been fully attained by any nation [4]. For instance, the case of ‘double burden of malnutrition’ (which goes beyond studying malnutrition as a separate health problem), is a condition where a child cohabits with undernutrition (such as stunting, and wasting) and overnutrition (such as overweight or obesity), is becoming a highly prevalent condition among children under-five years in Low and Middle Income Countries (LMICs) [5]. However, in the last two decades, stunting in children has witnessed significant reduction, while overweight is on the increase [3]. In Nigeria, the 2013 Nigeria Demographic and Health Survey (NDHS) reported that 37%, 18% and 29% of the children under-five years are stunted, wasted and underweight, respectively [4]. Understanding how different socio-economic, demographic, and contextual factors determine child malnutrition is a great way to improve the distribution of scare resources for the desired interventions. Variations also exist in the indicators of malnutrition as per the place of residence where urban children are likely to be more nourished than children in rural areas [6, 7]. To reach the nine targets, set forth by the WHO on malnutrition by 2025, researchers have committed to investigating malnutrition in its entirety, including the interconnection between undernutrition and overnutrition (particularly among children under the age of five years). A recent scoping review of predictors of malnutrition in its separate multifaceted form in SSA has identified both individual and household determining factors, and is reported elsewhere [8]. But, effective interventions cannot be created until a solid evidence base is built [9]. This study aims to assess the prevalence, and its individual and contextual predictors of malnutrition among children aged 6–59 months in Nigeria.

## Methods

### Source of data

This study is a secondary analysis of data from two separate, independently collected, nationally representative cross-sectional surveys. We took the Gender Inequality Index (GII), Human Development Index (HDI) and Multi-dimensional Poverty Index (MPI) out of the National Human Development Report (NHDR 2018) to use as the state variables and were included in the 2018 Nigeria Demographic and Health Survey (2018 NDHS), which is the primary data set. Using a two-stage stratified cluster design on each stratum obtained from the demarcation of the identified enumeration in the 2006 census, samples were independently chosen from each stratum in the NDHS. 1,400 Enumeration Areas (EAs) were chosen as sampling units at the first stage. At the second stage equal probability sampling was used to select 30 households at random from each EA, yielding a target sample size for the survey of 42,000 (30 × 1400) households. However, a weighted sample size of 7,770 children aged 6–59 months was used in the study.

### Variables definition

#### Outcome or dependent variable

In NDHS, the malnutrition status (MNS) of children under-five years was determined through the measurement of anthropometric indices expressed as (i) Stunting: measured as height-for-age Z-score (HAZ) of less than minus two standard deviations from the median of the reference population. It is an indicator of growth retardation. (ii) Wasting: a measure of weight-for-height Z-score (WHZ) of less than minus two standard deviations from the median of the reference population. (iii) Underweight: a measure of weight-for-age Z-score (WAZ) of less than minus two standard deviations from the median of the reference population [10, 11]; these are measures of undernutrition. (iv) Overweight is also a measure of weight-for-height Z-score (WHZ) of more than plus (+) two standard deviations above the median of the reference population [11], considered as a measure of ‘over-nutrition’. The measure of malnutrition was considered as the outcome of either undernutrition or over-nutrition indicators. A recumbent length for children less than two years old was taken lying down, while children two years and above had their height standing up using Shorr Board measuring instrument. On the other hand, the weights of the children were measured with the SECA scale (model 878U) [12]. In this study, the composite index for malnutrition was computed using the four indicators (stunting, wasting, underweight, and overweight) as a measure of the overall description of the ‘double burden of malnutrition’ status among children aged 6–59 months in Nigeria. Children with no trace of anthropometric failure were classified as ‘0’, labelled as ‘well nourished’, and those that have at least one of the four indicators were classified as ‘1’, labelled as ‘poorly nourished’ [13–15].

### Independent variables

The detailed descriptions and classifications of the predictor variables taken into consideration for this study have already been published elsewhere [8]. These variables were grouped into children/individual-related variables to include, child’s sex, age, perceived birth size, order, malaria status, nutritional status, fever, acute respiratory infection status, had diarrhoea, duration of breastfeeding, deworming, iron pills/syrup, and child took vitamin A; parental-related to include, place of delivery; preceding birth interval, maternal religious status, age group, age at first birth, educational status, working status, body mass index, anaemia status, autonomy level, marital status, ante-natal care visit, maternal ethnicity, religious status, maternal iron supplement during pregnancy, and paternal education status and work status; others are household-related to include, household socioeconomic status (wealth index), household size, number rooms for sleeping, number of under-five in the household, age and sex of household head, under-five slept under bed net last; and community-related variables to include, household region of residence, place of residence, community distance to health facility; and area-related variables to include, the state multidimensional poverty index, and the state human development index [8].

### Statistical analysis

At the first level of analysis, charts and spatial map were used to describe the prevalence of malnutrition. At the second level, multivariate multilevel logistic regression models were fitted to determine the predictors of malnutrition status among 6–59 months of age in Nigeria due to the complex/hierarchical nature of the data sets, where children/individuals (at level 1) are nested in communities/clusters (at level 2) and nested in states (at level 3). The 3-level model was found to be more appropriate than the 2-level model and single-level model using a likelihood ratio test. Stata 16 SE was used for the computations in this study. Variables were considered significant at 5% level; otherwise, they are stated on the table.

## Results

### Prevalence of malnutrition

**Fig. 1:**
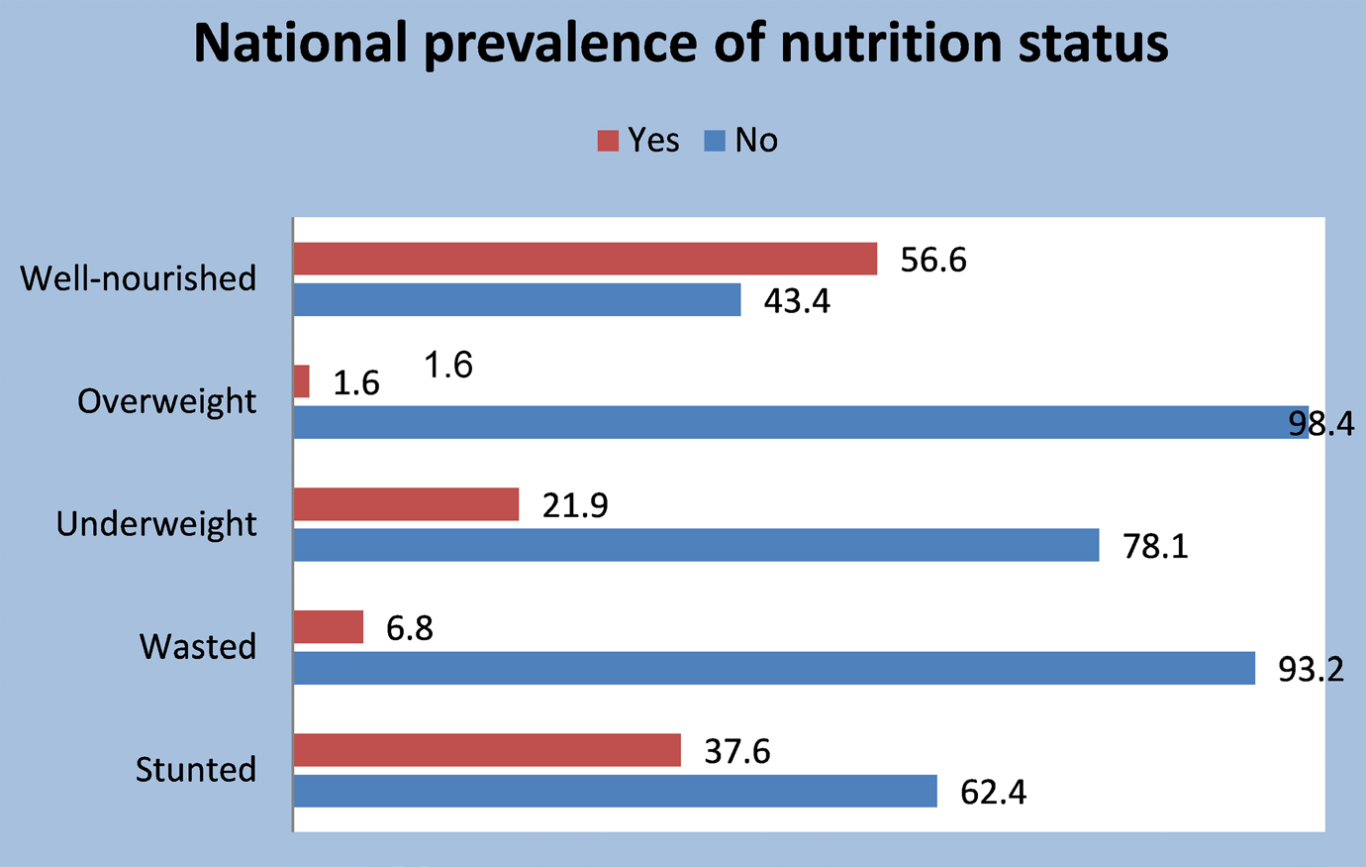
Prevalence of malnutrition status by indicators

Four indicators were used to establish the nutrition status of the children: stunting, wasting, underweight, and overweight. The ‘composite of anthropometric failure’ was used to classify the children as poorly nourished if the child had any indicators and represented as ‘1’, and well-nourished otherwise. Figure 1 indicates that 56.6% (5931/10,481), 95%CI (55.63–57.53) of the children is well nourished. In addition, about 38% (3942/10,481), 95%CI (36.7–38.5) of the children aged 6–59 months in Nigeria are stunted, 7% (709/10,481), 95%CI (6.30–7.26) are wasted, 22% (2296/10,481), 95%CI (21.12–22.71) are underweight, while about 2% (172/10,481), 95%CI (1.42–1.91) are overweight.

**Fig. 2:**
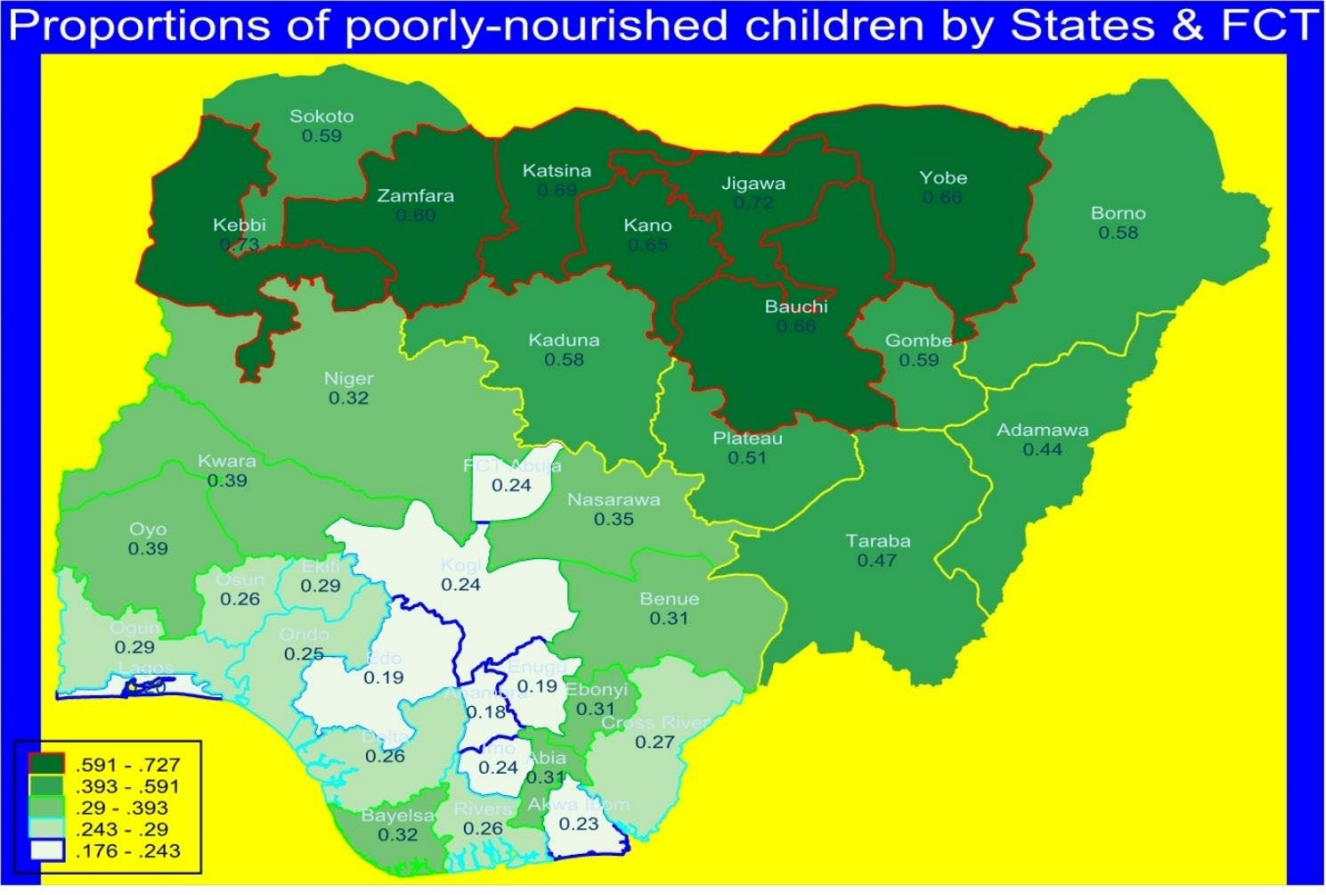
Spatial maps describing the proportions of poorly nourished children by states & FCT *Source*: Data computed from Nigeria DHS 2018.

Figure 2 presents the spatial variations in the proportion of children aged 6–59 months in Nigeria that were poorly nourished by states and Federal Capital Territory (FCT). The proportions of poorly nourished children were generally highest in the Northern Nigeria- Kebbi, Zamfara, Katsina, Kano, Jigawa, Bauchi and Gombe states. Edo and Lagos states are among the states with the least poorly nourished children.

### Multilevel analysis of malnutrition status

#### Multilevel multivariate models of predictors of malnutrition status

In the first instance, a multicollinearity test was conducted to check for highly correlated predictors. The outcome has already been reported elsewhere [16]. The backward and forward stepwise procedures were used to test all 48 variables that scaled through the multicollinearity checks. The removal was by p ≥ 0.20 for backward, and entry was *p <* 0.20 for stepwise forward methods, and then combined the outcomes of both forward and backward stepwise methods. Furthermore, the goodness of fits (Table 1) from these three methods using AIC and BIC shows that the combination of the backward and forward stepwise variables yielded the least AIC (9124.917).

**Table 1:** Evaluation of goodness of fit for variables selection methods in malnutrition

Goodness of fit	Backward stepwise	Forward stepwise	Backward + forward
AIC	9262.6	9154.846	9124.917
BIC	9729.8	9635.162	9639.811

Given above, 31 potential variables (child’s age, sex, birth size, preceding birth interval, child took iron syrup in the last six months before the survey, duration of breastfeeding, malaria status, anaemia status, cough status, diarrhoeal status, and child’s place of delivery, mother lives with partner, maternal education status, maternal ethnic group, maternal religious status, paternal education status, maternal body weight status, maternal anaemia status, household wealth, household head age group, number of bedrooms, low cluster wealth level, cluster distance to a health facility is no big problem, low cluster maternal education level, low cluster household with bed net, state human development index, state gender inequality index, region of residence, and place of residence), were finally retained for the multilevel model building.

### Model set-up

Given the complex/hierarchical nature of the data sets, such that children/parental/household in individual units at Level-1 (since children from the same parent, and household tend to be more similar than children from other households because they share the same characteristics) [16], are nested in communities/clusters at Level-2 and nested in states at Level-3, multivariate multilevel logistic regression models were fitted to determine the predictors of malnutrition status among 6–59 months of age in Nigeria. Furthermore, a likelihood ratio test was carried out to establish that the three-level model was more appropriate than the two-level model (the likelihood-ratio test is LR χ2 = 448.73, *p* < 0.001 for Level-2 nested in Level-3).

### Model building

The analysis built four multilevel logistic models. Model 1 is a null model (or empty model) with no predictors. The essence is to measure the variations across the communities and the states. Model 2 contains level-1 factors only. Model 3 contains level-1 and level-2 factors only (that is, community-related variables were added to Model 2); Model 4 (full model) was derived for all the selected variables, including the area-related variables (level-3 variables). Intra-class correlation coefficients (both the communities and the states), variance partition coefficients (both for the communities and states), and median odds ratios (MOR) were the post-estimation techniques used to evaluate the models. The goodness of fit was determined using Log-likelihood (LLH), Akaike’s information criteria (AIC) and Bayesian information criteria (BIC), such that the model with the highest LLH and lowest AIC and/or BIC was chosen as the best fit [16].

### Multilevel model results

This section presents the results from the analysis using multilevel mixed-effects logistic regression. A weighted total sample of 7770 children was analysed. These result into 1361 communities (level 2) with an average of 6 persons per community, nested in 37 states (level 3) with the number of children ranging between 92 and 373 and on average of 210 children per state.

### Measure of random effects

Model 1 is the null model (without predictors), and the random effect reveals that the proportion of the total variations attributable to differences in the communities and the states were, respectively, 0.322 (95%CI: 0.234 to 0.443) and 0.595 (95%CI: 0.367 to 0.966), while the variance attributable to individual level is 3.29 (2/3), which is fixed for logit. Therefore, the intrastate correlation coefficient of 0.1415 (95% CI: 0.092–0.210) and the intracommunity correlation coefficient of 0.218 (95% CI: 0.165–0.281) were used to evaluate the variations in the prevalence of malnutrition status among children aged 6–59 months in Nigeria due to the three-level factors. These results showed that respectively, state and community levels were responsible for 14.15% and 21.8% of the overall variation in the odds of malnutrition. As a result, the ICC and variance partition coefficient (VPC) at the state level are equivalent. The VPC at the community level, however, is 0.076, which means that a combined 7.6% of the overall variance is attributable to both the state and community levels.

AIC and likelihood ratios were used to evaluate the effectiveness of the models. Model 4 (complete model), with AIC = 9118.5 and log-likelihood = 4490.3, showed improvements in model fit. As a result, this study interpreted Model 4’s results. The ICC at the community level in the null model has decreased from 21.8 to 4.6% (95% CI: 17–25%) from the selected model (Model 4 in Table 2), meaning the correlation between two children/individuals (unit of analysis) within the same community and the same state is 0.046. The ICC at the state-level has also decreased from 14.1 to 0.1% (95% CI: 0.1–4.4%), both of which have maintained significance.

The variance partition coefficient (VPC) is the same as the ICC at the state level (0.002). Nevertheless, the VPC at the community level is 0.045, meaning that 4.5% of the total variance is collectively attributed to both the state and the community levels.

Similarly, in model 4, the median odds ratio (MOR) computed for states was 1.077, signifying that there exists a mean difference of the risk of being poorly nourished for two children with the same level-1 characteristics and picked randomly from two states. It means there is a 7.7% increased risk of a child being poorly nourished if he/she moves from one state to another state of increased risk of poorly nourished. Additionally, there is a 46% increased risk of a child being poorly nourished if he/she moves to another community with a higher risk of being poorly nourished.

### Measures of fixed effects

Table 2 displays the outcomes of the adjusted odds ratios (AOR) for the variables included in the analysis after accounting for the other factors in the model. The three levels of variables—individual variables at level 1, community variables at level 2, and state/area variables at level 3—were all included in Model 4, which was the model that best suited the data. Variables at level-1 are child’s age, sex, birth size, preceding birth interval; the child took iron syrup in the last six months before the survey, anaemia status, the child had diarrhoea in the last two weeks before the survey, mother currently works, maternal education status, maternal ethnicity, paternal education status, maternal body weight status, household wealth, and several bedrooms in the household were statistically significant predictors of malnutrition among children 6–59 months of age in Nigeria. Among the community-related variables (level 2-related), cluster distance to a health facility is no big problem and was a statistically significant predictor of malnutrition. Gender inequality index and region of residence among the area variables were also significant predictors of malnutrition. On the other hand, the child who took iron syrup in the last six months before the survey, the child’s duration of breast-feeding status, malaria RDT status, the child had cough two weeks before the survey, and place of delivery were not statistically significant predictors of malnutrition. Also, maternal religious status, maternal anaemia status, household head age group, number of people in the household, community wealth status, the proportion of community maternal education status, proportion of community households with no net, and type of place of residence, were not statistically significant predictors of malnutrition among children aged 6–59 months in Nigeria.

The odds of female children being poorly nourished (AOR = 0.74, 95% CI 0.67–0.82) are significantly lower than their male counterparts. Also, the odds of children aged 24–35 months developing malnutrition were 2.22 times the odds of children 6–11 months of age (AOR = 2.22, 95% CI: 1.72–2.86). The smaller the birth size of the children is, the more likely they will be poorly nourished. Children who were born with average birth size (AOR = 1.26, 95% CI: 1.05–1.51), and born with small birth size (AOR = 1.79, 95% CI: 1.45–2.26), had 26% and 79% increased odds of contracting malnutrition when compared with children born with large birth size. Anaemic children (AOR = 1.33, 95% CI: 1.18–1.51) and those who had diarrhoeal (AOR = 1.27, 95% CI: 1.08–1.49) two weeks preceding the survey were more prone to being poorly nourished compared with children who do not have any of these conditions.

**Table 2:** Multilevel multivariate logistic models of predictors of malnutrition with adjusted odds ratios (AOR) among children 6–59 months in nigeria

	Model 2 (N = 7770): Level-1 variables (child, parental, and households)		Model 3 (N = 7770): Level-1 & 2 variables (Model 2 + community-related)		Model 4 (N = 7770): Level 1, 2, & 3 variables (Model 3 + area-related)	
Variables	OR	95%CI	OR	95%CI	OR	95%CI
Child’s sex						
Male	1.00		1.00		1.00	
Female	0.742*	(0.669, 0.823)	0.743*	(0.669, 0.825)	0.742*	(0.669, 0.824)
**Child’s age in group**						
6–11 months	1.00		1.00		1.00	
12–23 months	1.69*	(1.402, 2.036)	1.688*	(1.4, 2.034)	1.664*	(1.381, 2.005)
24–35 months	2.282*	(1.771, 2.941)	2.268*	(1.759, 2.923)	2.219*	(1.722, 2.858)
36–47 months	1.848*	(1.428, 2.393)	1.847*	(1.426, 2.391)	1.791*	(1.384, 2.318)
48–59 months	1.318*	(1.014, 1.715)	1.312*	(1.008, 1.706)	1.276	(0.982, 1.659)
**Child’s birth size**						
Large	1.00		1.00		1.00	
Average	1.256*	(1.044, 1.512)	1.259*	(1.046, 1.515)	1.26*	(1.049, 1.514)
Small	1.805*	(1.428, 2.282)	1.809*	(1.431, 2.288)	1.79*	(1.419, 2.26)
**Preceding birth interval**						
None	1.00		1.00		1.00	
8–24 months	1.313*	(1.092, 1.579)	1.32*	(1.098, 1.587)	1.311*	(1.091, 1.575)
25–35 months	1.015	(0.85, 1.212)	1.019	(0.854, 1.217)	1.015	(0.85, 1.211)
36–59 months	0.839	(0.699, 1.008)	0.844	(0.703, 1.014)	0.841	(0.701, 1.011)
60 + months	0.753*	(0.591, 0.96)	0.758*	(0.594, 0.966)	0.757*	(0.594, 0.964)
**Took Iron supplements**						
No	1.00		1.00		1.00	
Yes	1.096	(0.948, 1.268)	1.099	(0.95, 1.27)	1.13	(0.979, 1.304)
**Duration of breastfeeding**						
Ever breastfed, not currently breastfeeding	1.00		1.00		1.00	
Never breastfed	1.169	(0.762, 1.793)	1.155	(0.753, 1.771)	1.185	(0.773, 1.815)
Still breastfeeding	0.996	(0.819, 1.211)	0.988	(0.813, 1.202)	0.974	(0.801, 1.184)
**Malaria RTD status**						
Negative	1.00		1.00		1.00	
Positive	1.078	(0.956, 1.216)	1.07	(0.949, 1.207)	1.071	(0.95, 1.207)
**Anaemia status**						
Not anaemic	1.00		1.00		1.00	
Anaemic	1.33*	(1.176, 1.505)	1.325*	(1.172, 1.499)	1.332*	(1.178, 1.506)
**Child had cough in last 2 weeks before the survey**						
No	1.00		1.00		1.00	
Yes	0.926	(0.801, 1.07)	0.932	(0.807, 1.077)	0.899	(0.779, 1.039)
**Child had diarrheal in last 2 weeks before the survey**						
No	1.00		1.00		1.00	
Yes	1.267*	(1.078, 1.49)	1.276*	(1.086, 1.501)	1.267*	(1.08, 1.488)
**Place of child’s delivery**						
Home	1.00		1.00		1.00	
Public facility	0.925	(0.801, 1.069)	0.933	(0.807, 1.079)	0.934	(0.809, 1.078)
Private facility	0.843	(0.689, 1.032)	0.843	(0.689, 1.033)	0.853	(0.698, 1.042)
Elsewhere	0.722	(0.475, 1.098)	0.717	(0.472, 1.091)	0.718	(0.473, 1.09)
**Mother/Caregiver currently working**						
No	1.00		1.00		1.00	
Yes	1.134	(0.997, 1.289)	1.128	(0.992, 1.282)	1.14*	(1.005, 1.294)
**Maternal/caregiver highest educational level**						
No education	1.00		1.00		1.00	
Primary	0.94	(0.786, 1.123)	0.938	(0.783, 1.123)	0.972	(0.812, 1.163)
Secondary	0.713*	(0.591, 0.859)	0.702*	(0.576, 0.854)	0.709*	(0.583, 0.863)
Higher	0.52*	(0.386, 0.701)	0.512*	(0.377, 0.695)	0.513*	(0.378, 0.696)
**Mother’s religious status**						
Catholic	1.00		1.00		1.00	
Other Christian	1.076	(0.861, 1.345)	1.093	(0.874, 1.367)	1.107	(0.891, 1.375)
Islam	1.206	(0.912, 1.594)	1.247	(0.942, 1.649)	1.203	(0.919, 1.575)
Traditionalist & others	0.868	(0.455, 1.656)	0.881	(0.462, 1.679)	1.029	(0.539, 1.966)
**Maternal ethnicity**						
Hausa/Fulani/Kanuri/Seribiri	1.00		1.00		1.00	
Ibos	0.542*	(0.382, 0.768)	0.544*	(0.383, 0.771)	0.633*	(0.415, 0.966)
Yoruba	0.879	(0.644, 1.201)	0.889	(0.651, 1.212)	1.187	(0.853, 1.651)
Others	0.737*	(0.599, 0.908)	0.733*	(0.595, 0.902)	0.831	(0.677, 1.021)
**Mother’s Anaemia status**						
Not Anaemic	1.00		1.00		1.00	
Anaemic	1.091	(0.977, 1.218)	1.088	(0.975, 1.215)	1.096	(0.983, 1.223)
**Maternal body weight status**						
Normal	1.00		1.00		1.00	
Underweight	1.267*	(1.064, 1.508)	1.267*	(1.064, 1.509)	1.266*	(1.063, 1.506)
Overweight	0.706*	(0.607, 0.82)	0.707*	(0.609, 0.822)	0.71*	(0.611, 0.824)
Obese	0.599*	(0.484, 0.741)	0.604*	(0.488, 0.748)	0.596*	(0.481, 0.737)
**Partner education status**						
No education	1.00		1.00		1.00	
Primary education	1.002	(0.829, 1.21)	1.003	(0.83, 1.213)	1.017	(0.842, 1.228)
Secondary education	0.96	(0.806, 1.143)	0.968	(0.812, 1.154)	0.965	(0.81, 1.148)
Tertiary education	0.789*	(0.629, 0.989)	0.797	(0.636, 1)	0.78*	(0.623, 0.977)
**Household wealth index**						
Poorest	1.00		1.00		1.00	
Poorer	0.895	(0.751, 1.067)	0.923	(0.773, 1.102)	0.943	(0.792, 1.122)
Middle	0.846	(0.698, 1.025)	0.952	(0.77, 1.177)	0.97	(0.787, 1.195)
Richer	0.639*	(0.516, 0.791)	0.764	(0.592, 0.986)	0.797	(0.62, 1.026)
Richest	0.571*	(0.438, 0.743)	0.687*	(0.508, 0.93)	0.732*	(0.543, 0.986)
**Household Head age group**						
Less 34 years	1.00		1.00		1.00	
35–44 years	0.945	(0.82, 1.089)	0.945	(0.819, 1.089)	0.946	(0.821, 1.091)
45–55 years	0.857	(0.72, 1.019)	0.861	(0.723, 1.024)	0.863	(0.725, 1.026)
56 years+	0.899	(0.736, 1.099)	0.913	(0.746, 1.116)	0.922	(0.755, 1.127)
**Number of bedrooms in household**						
One-room	1.00		1.00		1.00	
Two rooms	1.215*	(1.049, 1.408)	1.204*	(1.038, 1.395)	1.191*	(1.028, 1.38)
Three rooms	1.286*	(1.073, 1.541)	1.264*	(1.054, 1.515)	1.227*	(1.024, 1.469)
Four rooms	1.381*	(1.103, 1.729)	1.347*	(1.075, 1.689)	1.336*	(1.068, 1.673)
Five + rooms	1.207	(0.938, 1.553)	1.179	(0.916, 1.519)	1.158	(0.9, 1.49)
**Number of people in household**						
0–3	1.00		1.00		1.00	
4–6	0.836	(0.674, 1.036)	0.837	(0.675, 1.038)	0.84	(0.678, 1.041)
7–9	0.921	(0.718, 1.181)	0.921	(0.718, 1.182)	0.928	(0.724, 1.189)
10+	1.009	(0.761, 1.337)	1.013	(0.764, 1.344)	1.008	(0.761, 1.335)
**Community wealth level**						
Low			1.00		1.00	
High			0.797*	(0.657, 0.967)	0.845	(0.696, 1.026)
**Proportion of community distance to health facility is no big problem**						
Low			1.00		1.00	
High			0.918	(0.8, 1.054)	0.867*	(0.761, 0.988)
**Proportion of community maternal education level**						
Low			1.00		1.00	
High			1.103	(0.906, 1.344)	1.135	(0.933, 1.381)
**Proportion of community households with no bed net**						
Low			1.00		1.00	
High			0.935	(0.813, 1.076)	0.972	(0.848, 1.113)
**Gender inequality index by state (GII**)						
Lowest GII					1.00	
Low GII					1.454*	(1.144, 1.849)
Average GII					1.066	(0.819, 1.388)
High GII					0.814	(0.647, 1.023)
Highest GII					1.042	(0.783, 1.387)
**Region of residence**						
North-central					1.00	
North-east					2.272*	(1.773, 2.912)
North-west					3.111*	(2.339, 4.138)
South-east					1.106	(0.714, 1.712)
South-south					0.979	(0.734, 1.306)
South-west					1.093	(0.807, 1.481)
**Type of place of residence**						
Urban					1.00	
Rural					1.01	(0.869, 1.173)
**Intercept**	0.527	(0.319, 0.872)	0.547	(0.329, 0.91)	0.316*	(0.182, 0.547)
**Random effect**						
Community-level variance	0.166	0.098, 0.283	0.165	0.096, 0.282	0.155	0.089, 0.270
State-level variance	0112	0.057, 0.220	0.109	0.054, 0.219	0.006	0.000, 0.159
VPC: community-level	0.047		0.046		0.045	
VPC: state-level	0.031		0.031		0.002	
ICC: community-level	0.078		0.077		0.047	
ICC: state-level	0.031		0.031		0.002	
MOR: community	1.475	1.348, 1.661	1.473	1.344, 1.659	1.456	1.329, 1.641
MOR: state	1.376	1.256, 1.564	1.370	1.248, 1.563	1.077	1.014, 1.463
**Model fit statistic**						
**Log-likelihood**	-4520.5		-4516.16		-4490.25	
AIC	9150.996		9150.317		9118.499	
BIC	9533.688		9560.841		9598.603	

AOR: Adjusted Odds Ratios, ICC: Intraclass Correlation Coefficient, VPC: Variance Partition Coefficient, AIC: Akaike Information Criterion (Given a set of candidate models for the data, the preferred model is the one with the minimum AIC value)*p-value < 0.05

Similarly, children of working-class mothers have increased odds (AOR = 1.14, 95% CI: 1.01–1.29) of being poorly nourished compared with children whose mothers do not work. The educational levels attained by mothers and fathers are inversely proportional to the odds of their children being poorly nourished. Children of mothers with secondary education (AOR = 0.71, 95% CI: 0.58–0.86), tertiary education (AOR = 0.51, 95% CI: 0.38–0.70), and fathers with tertiary education (AOR = 0.78, 95% CI: 0.62–0.98), were respectively, 29%, 49%, and 22% reduced odds of being poorly nourished compared with children whose mothers and fathers do not have any formal education. The results also show that the richer the household, the less likely the children will be poorly nourished compared with their counterparts in the poorest household. Also, the odds of children residing in a 3-bedroom household (AOR = 1.23, 95% CI: 1.02–1.47) are 1.23 times more likely to be poorly nourished than children residing in a one-bedroom household. Furthermore, among the community-related variables, children from a community where the proportion of the community distance to the nearest health centre is not a big problem are high; there are 17% reduced odds of being poorly nourished compared with children from a community with a low proportion. Moreover, from the area-related variables, the odds of children from a state where the gender inequality index is low (AOR = 1.45, 95% CI: 1.14–1.85) is significantly 1.45 times more likely to be poorly nourished when compared with children from the state with lowest gender inequality index. The odds of children from the North-east (AOR = 2.27, 95% CI: 1.77–2.94), and the North-west (AOR = 3.11, 95% CI: 2.40–4.14), are 127% and 211% significantly more likely to be poorly nourished respectively.

## Discussion

In the random effect analysis, the intrastate correlation coefficient of 0.142 and the intracommunity correlation coefficient (ICC) of 0.218 were used to assess the variations in malnutrition status among children aged 6–59 months in Nigeria due to the three-level factors. These results showed that 14.2% and 21.8% of the total variation in the odds of malnutrition were due to state and community levels, respectively. In addition, the ICC at the state level also decreased from 14.1 to 0.1%, which was statistically significant. Furthermore, the ICC at the community level in the null model decreased from 21.8 to 4.6% in the choice model, meaning the correlation between two children/individuals (unit of analysis) within the same community and the same state is 0.046. Similarly, model 4’s median odds ratio (MOR) calculated for states was 1.077, indicating that if a child travels from one state to another there is a greater chance of 7.7% of increased risk of being poorly nourished. In addition, moving to a community with a higher risk of malnutrition increases a child’s probability of being poorly nourished by 46%.

In the fixed effect analysis, the study found that female children have considerably decreased odds of being poorly nourished than their male counterparts. The same findings were reported in previous studies on malnutrition [8, 17–30], The risks of children aged 24–35 months cohabiting malnutrition were 2.22 times higher than those of children aged 6–11 months. Similar conclusions were reached in previous studies that an increase in the age of the children has a harmful effect on poor nourishment for the children [31–43]. The possible explanation for this difference is that as the child grows older, less attention is paid to their welfare compared with the youngest child. Also, children who had diarrhoea two weeks before the survey were more likely to be poorly nourished than children who did not have the illnesses. This finding is supported by previous studies [32, 33, 43–46]. Similarly, children of working-class mothers are more likely to be poorly nourished than children whose mothers do not work. This agrees with another study [47]. The possible reason for this is that working class mothers do not breast feed their children much longer compared to mothers who do not work. The odds of being poorly nourished are inversely proportional to the educational status that mothers and fathers have obtained. Other studies substantiated the result on maternal education [31–34, 36, 38–40, 42, 44–46, 48–52], and for paternal educational attainment was supported in [33, 39, 51]. The results also show that the richer the household, the less likely the children from such a home will be poorly nourished compared with their counterparts from the poorest household. This finding agrees with other studies [31–34, 36–40, 42–46, 48, 50–54], and contrary finding with [55].

In addition, children from communities where many residents do not consider distance to the nearest health facility to be ‘no big problem’ are 17% less likely to be poorly nourished than children from communities with a low proportion do exist. The odds of children from a state with a low gender inequality index being poorly nourished are significantly 1.45 times higher than those with the highest gender inequality index. The odds of children from the North-east, and the North-west, are 127% and 211%, significantly more likely to be poorly nourished, respectively. Children living in urban areas are more protected from being malnourished than children from rural areas. This result agrees with other previous studies [36, 38, 42, 44, 46, 48, 50, 51].

### Strengths and limitations of the study

This study is based on the analysis of the merged data sets whereby contextual variables from 2018 NHDR were incorporated into the 2018 NDHS. Moreover, the study took into consideration the hierarchical nature of the data set and conducted a 3-state multilevel mixed effect logistic analysis to account for individual, community, and state variabilities. In addition, the study recognised overweight as part of the indicators (stunting, wasting, and underweight) that formed the proxies for malnutrition, otherwise known as the index of double burden of malnutrition.

There are several restrictions on this study. First, because the survey was cross-sectional, the study could only look at the relationships between factors. Consequently, it was impossible to determine causality. It’s also important to note that the random coefficient model in a three-level model was not examined in this study to see if it was superior to the variance component and random intercept model. Additionally, only the individual level of weighting was considered. These are appropriate for further research.

## Conclusion

In general, the Northern Nigerian states of Kebbi, Zamfara, Katsina, Kano, Jigawa, Bauchi, and Gombe had the highest proportions of poorly malnourished children aged 6–59 months. Most of these areas are highly affected by high rates of constant insecurities that have distorted farming activities [56]. Efforts should be made to end these age long insecurities in these areas. The results reported female children are well-nourished compared to their male counterparts. Therefore, gender-sensitive policies and community mobilisation against gender-based bias are required to overcome the discrepancies in malnutrition status between male and female child.

## Data Availability

The data set used in this study is available in MeasureDHS https://dhsprogram.com (accessed on 28 January 2020) and UNDP-Nigeria https://hdr.undp.org/content/national-human-development-report-2018-nigeria (accessed on 3 March 2020).

## Abbreviations

LMICs low: and middle-income countries
WHO: World Health Organization
NDHS: Nigerian Demographic and Health Survey
NHDR: Nigeria Human Development Report
GII: Gender Inequality Index
HDI: Human Development Index
MPI: Multidimensional Poverty Index
EAs: Enumeration Areas
MNS: Malnutrition Status
HAZ: Height for Age Z-score
WHZ: Weight for Height Z-score
WHO: World Health Organization
WAZ: Weight for Age Z-score
CI: Confidence Interval
FCT: Federal Capital Territory
AIC: Akaike’s Information Criteria
BIC: Bayesian Information Criteria
LR: Likelihood Ratio
MOR: Median Odds Ratio
LLH: Log-Likelihood
ICC: Intra-Class Correlation
AOR: Adjusted Odds Ratio
RDT: Rapid Diagnostic Test
TETFUND: Tertiary Education Trust Fund

## References

[1] World Health Organisation. Fact sheets - Malnutrition, https://www.who.int/news-room/fact-sheets/detail/malnutrition (2020, accessed 31 May 2020).

[2] Endris N, Asefa H, Dube L. Prevalence of Malnutrition and Associated Factors among Children in Rural Ethiopia. *Biomed Res Int* 2017; 2017: 6587853. 10.1155/2017/6587853 PMC5449753 28596966

[3] Global Nutrition Report. The burden of malnutrition, https://globalnutritionreport.org/reports/global-nutrition-report-2018/burden-malnutrition/ (2020, accessed 25 June 2020).

[4] National Population Commission (NPC). ICF International. Nigeria Demographic and Health Survey 2013. Federal Republic of Nigeria and MeasureDHS; June 2014.

[5] Editorial. A future direction for tackling malnutrition. The Lancet Vol 395(027), p2, January 04, 2020. 10.1016/S0140-6736(19)33099-5. 10.1016/S0140-6736(19)33099-5 31852604

[6] Babatunde RO, Olagunju FI, Fakayode SB (2011). Prevalence and determinants of Malnutrition among under-five Children of Farming Households in Kwara State, Nigeria. JAS.

[7] National Population C. I. C. F. International. Nigeria Demographic and Health Survey 2003. *Federal Republic of Nigeria and MeasureDHS*.

[8] Obasohan PE, Walters SJ, Jacques R et al. Risk Factors Associated with Malnutrition among Children Under-Five Years in Sub-Saharan African Countries: A Scoping Review. *International Journal of Environmental Research and Public Health* 2020; 17: 8782. 10.3390/ijerph17238782 PMC7731119 33256022

[9] Kwansa AL, Akparibo R, Cecil JE,. Risk Factors for Overweight and Obesity within the Home Environment of Preschool Children in Sub-Saharan Africa: A Systematic Review. *Nutrients* 2022; 14: 1706. *LMU* 2011; 18. 10.3390/nu14091706 PMC9100775 35565675

[10] Kandala NB, Lang S, Klasen S et al. Semiparametric Analysis of the Socio-Demographic and Spatial Determinants of Undernutrition in Two African Countries. *LMU* 2011; 18.

[11] National Population C, International ICF (2019). Nigeria Demographic and Health Survey 2018.

[12] National Population Commission. (NPC)[Nigeria], ICF. Nigeria Demographic and Health Survey; 2018.

[13] Bamiwuye SO, Wet ND, Adedini SA (2013). Linkages between autonomy, poverty and contraceptive use in two sub-saharan African countries. Afr Popul Stud.

[14] Nandy S, Daoud A, Gordon D. Examining the changing profile of undernutrition in the context of food price rises and greater inequality. Soc Sci Med. 2016;149:153–63. 10.1016/j.socscimed.2015.11.036 26723002

[15] Nandy S, Jaime Miranda J (2008). Overlooking undernutrition? Using a composite index of anthropometric failure to assess how underweight misses and misleads the assessment of undernutrition in young children. Soc Sci Med.

[16] Obasohan PE, Walters SJ, Jacques R (2021). Individual and Contextual Factors Associated with Malaria among children 6–59 months in Nigeria: a multilevel mixed Effect Logistic Model Approach. Int J Environ Res Public Health.

[17] Adekanmbi VT, Uthman OA, Mudasiru OM (2013). Exploring variations in childhood stunting in Nigeria using league table, control chart and spatial analysis. BMC Public Health.

[18] Akombi BJ, Agho KE, Hall JJ, et al. Stunting and severe stunting among children under-5 years in Nigeria: a multilevel analysis. BMC Pediatr. 2017;17:15. 10.1186/s12887-016-0770-z PMC5237247 28086835

[19] Akombi BJ, Agho KE, Renzaho AM (2019). Trends in socioeconomic inequalities in child undernutrition: evidence from Nigeria Demographic and Health Survey (2003–2013). PLoS ONE.

[20] Amaral MM, Herrin WE, Gulere GB (2017). Using the Uganda National Panel Survey to analyze the effect of staple food consumption on undernourishment in Ugandan children. BMC Public Health.

[21] Amare ZY, Ahmed ME, Mehari AB. Determinants of nutritional status among children under age 5 in Ethiopia: further analysis of the 2016 Ethiopia demographic and health survey. Globalization and Health; 15. Epub ahead of print 2019. 10.1186/s12992-019-0505-7. 10.1186/s12992-019-0505-7 PMC6836473 31694661

[22] Fantay Gebru K, Mekonnen Haileselassie W, Haftom Temesgen A et al. Determinants of stunting among under-five children in Ethiopia: a multilevel mixed-effects analysis of 2016 Ethiopian demographic and health survey data. BMC Pediatr; 19. Epub ahead of print 2019. 10.1186/s12887-019-1545-0. 10.1186/s12887-019-1545-0 PMC6544992 31153381

[23] Hv D, S N-S (2017). Trends and determinants of child growth indicators in Malawi and implications for the Sustainable Development Goals. AIMS Public Health.

[24] Machisa M, Wichmann J, Nyasulu PS (2013). Biomass fuel use for household cooking in Swaziland: is there an association with anaemia and stunting in children aged 6–36 months?. Trans R Soc Trop Med Hyg.

[25] Magadi MA (2011). Household and community HIV/AIDS status and child Malnutrition in sub-saharan Africa: evidence from the demographic and health surveys. Soc Sci Med.

[26] Miller CM, Gruskin S, Subramanian SV (2007). Emerging health disparities in Botswana: examining the situation of orphans during the AIDS epidemic. Soc Sci Med.

[27] Nankinga O, Kwagala B, Walakira E. Maternal employment and child nutritional status in Uganda, https://www.ncbi.nlm.nih.gov/pmc/articles/PMC6922416/ (2019, accessed 11 August 2020). 10.1371/journal.pone.0226720 PMC6922416 31856209

[28] Nshimyiryo A, Hedt-Gauthier B, Mutaganzwa C (2019). Risk factors for stunting among children under five years: a cross-sectional population-based study in Rwanda using the 2015 demographic and Health Survey. BMC Public Health.

[29] Takele K, Zewotir T, Ndanguza D (2019). Understanding correlates of child stunting in Ethiopia using generalized linear mixed models. BMC Public Health.

[30] Ukwuani FA, Suchindran CM (2003). Implications of women’s work for child nutritional status in sub-saharan Africa: a case study of Nigeria. Soc Sci Med.

[31] Aboagye RG, Seidu AA, Ahinkorah BO et al. Dietary Diversity and Undernutrition in Children Aged 6–23 Months in Sub-Saharan Africa. *Nutrients*; 13. Epub ahead of print 28 September 2021. 10.3390/nu13103431. 10.3390/nu13103431 PMC8537414 34684435

[32] Adam Birhan N, Bitew Belay D (2021). Associated risk factors of underweight among under-five children in Ethiopia using multilevel ordinal logistic regression model. Afr H Sci.

[33] Bekele SA, Fetene MZ (2021). Modeling non-gaussian data analysis on determinants of underweight among under five children in rural Ethiopia: Ethiopian demographic and health survey 2016 evidences. PLoS ONE.

[34] Kassie GW, Workie DL (2020). Determinants of under-nutrition among children under five years of age in Ethiopia. BMC Public Health.

[35] Kebede D, Aynalem A (2021). Prevalence of undernutrition and potential risk factors among children below five years of age in Somali region, Ethiopia: evidence from 2016 Ethiopian demographic and health survey. BMC Nutr.

[36] Khamis AG, Mwanri AW, Kreppel K (2020). The burden and correlates of childhood undernutrition in Tanzania according to composite index of anthropometric failure. BMC Nutr.

[37] Masibo PK, Humwa F, Macharia TN (2020). The double burden of overnutrition and undernutrition in mother – child dyads in Kenya: demographic and health survey data, 2014. J Nutr Sci.

[38] Muche A, Dewau R (2021). Severe stunting and its associated factors among children aged 6–59 months in Ethiopia; multilevel ordinal logistic regression model. Ital J Pediatr.

[39] Rutayisire R, Kanazayire C, Tuyisenge G (2020). Trends in the prevalence and Associated contributing factors of stunting in children under five years of age. Secondary Data Analysis of 2005, 2010 and 2014–2015 Rwanda demographic and health surveys. RJMHS.

[40] Simelane MS, Chemhaka GB, Zwane E (2020). A multilevel analysis of individual, household and community level factors on stunting among children aged 6–59 months in Eswatini: a secondary analysis of the Eswatini 2010 and 2014 multiple Indicator Cluster surveys. PLoS ONE.

[41] Sserwanja Q, Mutisya LM, Olal E et al. Factors associated with childhood overweight and obesity in Uganda: a national survey. *Bmc Public Health*; 21. Epub ahead of print August 2021. 10.1186/s12889-021-11567-1. 10.1186/s12889-021-11567-1 PMC8330108 34344336

[42] Tesema GA, Yeshaw Y, Worku MG (2021). Pooled prevalence and associated factors of chronic undernutrition among under-five children in East Africa: a multilevel analysis. PLoS ONE.

[43] Uwiringiyimana V, Osei F, Amer S (2022). Bayesian geostatistical modelling of stunting in Rwanda: risk factors and spatially explicit residual stunting burden. BMC Public Health.

[44] Chikako TU, Seidu A-A, Hagan JE (2021). Complex Multilevel Modelling of the Individual, Household and Regional Level variability in predictors of undernutrition among children aged 6–59 months in Ethiopia. Nutrients.

[45] Muche A, Gezie LD, Baraki AG (2021). Predictors of stunting among children age 6–59 months in Ethiopia using bayesian multi-level analysis. Sci Rep.

[46] Tesfaw LM, Fenta HM (2021). Multivariate logistic regression analysis on the association between anthropometric indicators of under-five children in Nigeria: NDHS 2018. BMC Pediatr.

[47] Amegbor PM, Yankey O, Sabel CE (2020). Examining the Effect of Geographic Region of Residence on Childhood Malnutrition in Uganda. J Trop Pediatr.

[48] Adedokun ST, Yaya S (2021). Factors associated with adverse nutritional status of children in sub-saharan Africa: evidence from the Demographic and Health Surveys from 31 countries. Matern Child Nutr.

[49] Amaha ND, Woldeamanuel BT. Maternal factors associated with moderate and severe stunting in Ethiopian children: analysis of some environmental factors based on 2016 demographic health survey. Nutr J. 2021;20:18. 10.1186/s12937-021-00677-6 PMC7916293 33639943

[50] Amoako Johnson F (2022). Spatiotemporal clustering and correlates of childhood stunting in Ghana: analysis of the fixed and nonlinear associative effects of socio-demographic and socio-ecological factors. PLoS ONE.

[51] Fenta HM, Zewotir T, Muluneh EK. Spatial data analysis of malnutrition among children under-five years in Ethiopia. *Bmc Medical Research Methodology*; 21. Epub ahead of print October 2021. 10.1186/s12874-021-01391-x. 10.1186/s12874-021-01391-x PMC8549278 34706661

[52] Hailu BA, Bogale GG, Beyene J (2020). Spatial heterogeneity and factors influencing stunting and severe stunting among under-5 children in Ethiopia: spatial and multilevel analysis. Sci Rep.

[53] Amegbor PM, Zhang Z, Dalgaard R (2020). Multilevel and spatial analyses of childhood Malnutrition in Uganda: examining individual and contextual factors. Sci Rep.

[54] Musuka GN, Dzinamarira T, Cuadros DF (2021). Mothers’ HIV status and their children’s nutritional status: insights from secondary analysis of the Zimbabwe Demographic and Health Survey data (2015–2016). Food Sci Nutr.

[55] Wondimu H, Dejene K (2022). Determinants of under-five Malnutrition, significant changes, and policy implications in the Ethiopian Demographic Health Survey, 2019. Discov Sustain.

[56] Fudjumdjum H, Filho WL. Ayal DY Assessment of Barriers To Food Security in North-Eastern Nigeria. Handb Clim Change Resil. Aug 2019;13:1019–33.

